# Physicochemical characterization and antioxidant activity of polysaccharides from *Chlorella* sp. by microwave-assisted enzymatic extraction

**DOI:** 10.3389/fbioe.2023.1264641

**Published:** 2023-08-10

**Authors:** Hao Peng, Xiangjin Xv, Xiangwei Cui, Yongxiang Fu, Shuqi Zhang, Guanhao Wang, Xue Chen, Wenlu Song

**Affiliations:** ^1^ New Energy Research Institute, Jining University, Jining, China; ^2^ School of Life Sciences, Yunnan University, Kunming, China

**Keywords:** *Chlorella* sp., polysaccharides, response surface methodology, microwave-assisted enzymatic extraction, physicochemical characterization

## Abstract

Microwave-assisted enzymatic extraction (MAEE) was used for the separation of polysaccharides from micro-*Chlorella*. The extraction condition of MAEE was optimized by Box-Behnken design and response surface methodology. Results showed that the optimal condition for the extraction of *Chlorella* sp. crude polysaccharides (CSCP) was at 50°C for 2.3 h with 380 W of microwave power and 0.31% of enzyme dosage. Under the optimal extraction condition, the extraction yield of CSCP reached 0.72%. Similarly, the α-amylase modification conditions of the CSCP were also optimized, in which the 1,1-diphenyl-2-picrylhydrazyl (DPPH) radical scavenging rate was used as the response value. The scavenging rate of DPPH free radicals was 17.58% when enzyme dosage was 271 U/g at 51°C for 14 min. Moreover, the enzyme-modified CSCP presented a typical heteropolysaccharide mainly including glucose (48.84%), ribose (13.57%) and mannose (11.30%). MAEE used in this work achieved a high extraction yield of CSCP, which provides an efficient method for the extraction of CSCP from *Chlorella* sp.

## 1 Introduction

In Eustigmatophyceae, many fast-growing single-cell microalgae are usually named as Nannochloropsis. Microcystis presents the property of fast growth rate, high photosynthetic efficiency, low planting cost, and environmentally friendly (Zhang and Liu, 2021; Sarwer et al., 2022). Microalgae are rich in protein, pigment, oil and various polyunsaturated fatty acids, especially eicosapentaenoic acid (Kratzer and Murkovic, 2021). The downstream products of the microalgae can be used as ideal raw materials for the production of animal feed, food additives, human health food, cosmetics, biofuels, *etc.* (Siddiki et al., 2022; Sivaramakrishnan et al., 2022). In recent years, more and more attention has been paid to microalgae polysaccharides due to its excellent characteristics, such as antioxidant, antiproliferative, hypoglycemic, and adjuvant activities (Freitas et al., 2021; Mohammed et al., 2021).

Until now, many methods have been explored and innovated for the extraction of microalgae polysaccharides including hot water extraction (HWE), microwave-assisted extraction (MAE), enzyme-assisted extraction (EAE) and ultrasonic-assisted extraction (UAE) (Lin et al., 2022). Although the HWE method has the advantages of low cost, nontoxicity, and safe operation, it has been limited in practical application due to the disadvantages of long extraction time and low extraction rate (Wang et al., 2023). Comparatively, MAE has been considered an effective method for its high extraction rate, low energy consumption and good preservation of polysaccharide biological activity. It has been reported that polysaccharides extracted by MAE method possess a higher sulfate groups concentration and a lower molecular weight, resulting in the high hydroxyl radical scavenging activity and antioxidant of the polysaccharides (Ren et al., 2017; Okolie et al., 2019). Sulfated polysaccharides from green tide algae *Ulva prolifera* could also be effectively extracted by microwave assisted hydrothermal extraction technology (Yuan et al., 2018). In addition, as compared to HWE method, the monosaccharide compositions and the type of glycosidic bond in fucoidan were almost unaffected when extracted by MAE method from *Sargassum thunbergii* (Ren et al., 2017). Cao et al. (2018) reported that polysaccharides extraction yield from *Sargassum pallidum* increased significantly with increased time, temperature and power, while optimal MAE conditions were set at 10 min, 90°C, and 800 W. However, the extraction rate from MAE method was also limited due to the stubborn cell wall structure of the microalgae.

The application of various coordinated extraction technologies, such as microwave-assisted enzymatic extraction (MAEE) and ultrasound-assisted enzymatic extraction (UAEE), greatly make up for the defects of single extraction method to a certain extent. EAE method, with the characteristics of low energy consumption and fast extraction rate, has been developed for the catalytic hydrolysis of polysaccharides (Nadar et al., 2018; Wang and Li, 2022; Jin et al., 2023). The use of enzymes capable of degrading the cell wall would promote the degradation of polysaccharides into smaller fragments, facilitating the subsequent extraction. Generally, carbohydrate hydrolytic enzymes, proteases and pectinase were applied for the degradation of cell wall to promote the release of intracellular contents in cell wall. Alboofetileh et al. (2019) reported that the fucoidan yields from UAE and EAE method were 3.6% and 5.6%, respectively, while the combination of UAE and EAE yielding the higher fucoidan yield of 7.9%. MAEE method had been developed to enhance the extraction of crude polysaccharides from the fruit of *Schisandra chinensis* baill. The optimum extraction yield was 7.38% with microwave irradiation time of 10 min and enzyme concentration of 1.5% at 47.58°C for 3 h (Cheng et al., 2015).

Polysaccharides exist in organisms and have been widely used in feed, food and medicine. However, its weak solubility and dispersion in various media have seriously limited its further application (Halahlah et al., 2022). Therefore, researchers have made many modifications to change its spatial structure, presenting the desired physical, chemical and biological properties. The biological activity of the polysaccharides could be significantly enhanced by the shear modification of spatial structure of the polysaccharides. Research reported that enzyme modification could change the basic structure and intermolecular force of polysaccharides, resulting in conformational transformation in solution, which can directly affect its biological activity (Hua et al., 2022). It was reported that the improvement of biological activity has been one of the bottlenecks of the complex structure of glycans (Ma et al., 2020). The complex macromolecules of glycans could be depolymerized into accurate small structural units by enzymatic hydrolysis to achieve better biological activity (Jönsson et al., 2020). Hence, it is of great significance to explore effective methods for the extraction and modification of the polysaccharides from microalgae, which would provide a basis for further research and wide application of the microalgae.

The focus of the present study was to systemically probe the effects of different extraction condition on the *Chlorella* sp. crude polysaccharides (CSCP) yields. MAEE method was used for the extraction of polysaccharides from *Chlorella* sp. The optimal extraction parameters, α-amylase modification conditions, and physicochemical properties of CSCP were investigated. The extraction condition of MAEE was optimized by Box-Behnken design (BBD) and response surface methodology (RSM). The polysaccharides extraction rate was used as the response value, while the three factors of enzyme dosage, microwave power and extraction time were selected as the independent variables. Similarly, the α-amylase modification conditions of the target polysaccharides from microalga *Chlorella* sp. were also optimized, in which the enzyme dosage, temperature and hydrolysis time were selected as the independent variables and the 1,1-diphenyl-2-picrylhydrazyl (DPPH) free radical scavenging rate was used as the response value. Finally, Design-Expert software was applied to optimize the extraction condition and enzyme modification condition of microalgae polysaccharides.

## 2 Materials and methods

### 2.1 Materials and chemical reagents

The microalgae (*Chlorella* sp.) used in the experiments were cultivated from the New Energy Research Institute, Jining University. The freeze-dried samples were grinded to screen out the 40–60 mesh fraction. All chemicals were analytical grade and purchased from Sigma-Aldrich.

### 2.2 Extraction process of the polysaccharides

*Chlorella* sp*.* powder (10 g) was dispersed in 100 mL of distilled water and then the mixture was placed in an extractor to perform microwave treatment at 50°C for 15 min with different microwave power (150, 250, 350, 450, and 550 W). Then, different enzyme dosage (0.1%, 0.2%, 0.3%, 0.4%, and 0.5%, cellulase: pectinase = 1:1) was added to the solution to extract for 1.0, 1.5, 2.0, 2.5, and 3.0 h, respectively. After extraction process, the enzyme was inactivated in a water bath at 80°C for 30 min, cooled and centrifuged in a high-speed refrigerated centrifuge at 5,000 r/min and 4°C for 15 min to get the supernatant. After the supernatant was distilled under reduced pressure, alcohol-precipitated polysaccharide was carried out with 3 times the volume of the remaining solution volume of absolute ethanol, and it was allowed to stand for 10 min. Finally, after alcohol precipitation, the mixed solution was centrifuged at 5,000 r/min and 4°C for 15 min to collect the precipitated polysaccharides.

### 2.3 RSM design for optimization of polysaccharides extraction

Based on single-factor experiments, the preliminary range of enzyme dosage, microwave power and extraction time was determined, RSM was employed for optimization of *Chlorella* sp. crude polysaccharides (CSCP) extraction condition. The extraction experiment was conducted with 3 independent variables at 3 levels and is depicted in Table 1.

**TABLE 1:** Independent variables and their levels used for CSCP extraction.

Independent variables		Factor level		
		−1	0	1
A	Enzyme dosage (%)	0.2	0.3	0.4
B	Microwave power (W)	250	350	450
C	Extraction time (h)	1.5	2.0	2.5

### 2.4 Enzyme modification of the CSCP

The protein and pigment in the extracted CSCP were removed by the Sevage method and activated carbon method, respectively (Huang and Huang, 2020). Briefly, powdered activated carbon with the concentration of 20 mg/mL was added into the polysaccharides solution (20 mL), and then the mixture was incubated at 45°C for 1 h with shaking at 150 rpm. After centrifugation, the supernatant was collected for protein removal. Sevage reagent was prepared by mixing chloroform and n-butanol in a ratio of 3:1. The decolorized sample solution was mixed with Sevage reagent at a ratio of 4:1, stirred with slurry mixer for 0.5 h, and then refrigerated centrifugation for 10 min at 4°C with shaking at 10,000 rpm. The above operation was repeated 4 times, the supernatant was concentrated and freeze-dried for further analyzed.

The enzyme modification was conducted by adding distilled water and α-amylase (240 U/g) into the CSCP solution with a concentration of 1 mg/mL. Afterwards, a temperature gradient was set at the modification temperature of 40–60°C, and the CSCP was modified for 15 min. The effect of enzyme dosage was also explored at 50°C for 15 min with different enzyme concentrations of 160, 200, 240, 280, and 320 U/g. In addition, different modification time was also evaluated at 50°C with 240 U/g enzyme dosage for 5, 10, 15, 20, and 25 min, respectively. After the reaction, the enzyme was inactivated in a boiling water bath for 10 min to obtain the enzyme-modified CSCP.

### 2.5 RSM design for optimization of enzyme modification conditions

The experimental design was based on the results of the single factor test. As shown in Table 2, three factors of enzyme dosage, hydrolysis time, and modification temperature were independent variables, and the DPPH radical scavenging rate was set as the response value.

**TABLE 2:** Independent variables and their levels used for the enzyme modification of CSCP.

Independent variables		Factor level		
		−1	0	1
A	α-amylase dosage (U/g)	200	240	280
B	Hydrolysis time (min)	10	15	20
C	Temperature (^o^C)	45	50	55

### 2.6 Analytical methods

#### 2.6.1 Determination of polysaccharide extraction rate

In this experiment, the phenol-sulfuric acid polysaccharide detection method was used to detect the absorbance value of the crude CSCP and calculate the extraction rate.

$$Polysaccharide\;extraction\;rate=\frac{Quality\;of\;polysaccharide\;from\;microalgae}{Quality\;of\;microalgae\;powder}\times 100$$ (1)

The monosaccharide compositions of crude CSCP and enzyme-modified CSCP were detected via the high-performance liquid chromatography (HPLC; LC-20AD) using C18 (4.6 mm × 200 mm) column and RID. The operating temperature of the detector and column was 30°C. Additionally, Fourier-transform infrared spectroscopy (FT-IR) and molecular weight of the polysaccharides were carried out according to the previous reports (Ma et al., 2021; Wang et al., 2022).

#### 2.6.2 Enzyme modification index detection method

The DPPH radical scavenging rate was used as the detection index of the modification effect of CSCP. DPPH solution (2 mL) with a concentration of 0.1 mmol/L was added to 2 mL of the prepared enzyme-modified polysaccharide solution (0.1 mg/mL). After mixing, the reaction was kept in the dark at room temperature for 30 min. Then, the absorbance value Ai was measured at 517 nm. Enzyme-modified polysaccharide solution (2 mL) was added to 2 mL of absolute ethanol, and the processing operation was the same as above. The absorbance value Aj was measured. DPPH solution (2 mL) was added to 2 mL of absolute ethanol, and the absorbance value Ao was measured. The mixed solution of 2 mL of distilled water and 2 mL of anhydrous ethanol follows the same process as above for zero adjustment in this test process (Yan et al., 1998; Marinova and Batchvarov, 2011). The formula for calculating the DPPH radical scavenging rate (Sa/%) is as follows:

$$Sa\;\left(\%\right)=\left(1-\frac{Ai-Aj}{Ao}\times 100\right)$$ (2)

## 3 Results and discussion

### 3.1 Single-factor experiments and optimization of extraction conditions

#### 3.1.1 Effects of enzyme dosage, microwave power and extraction time on CSCP yield

**FIGURE 1:**
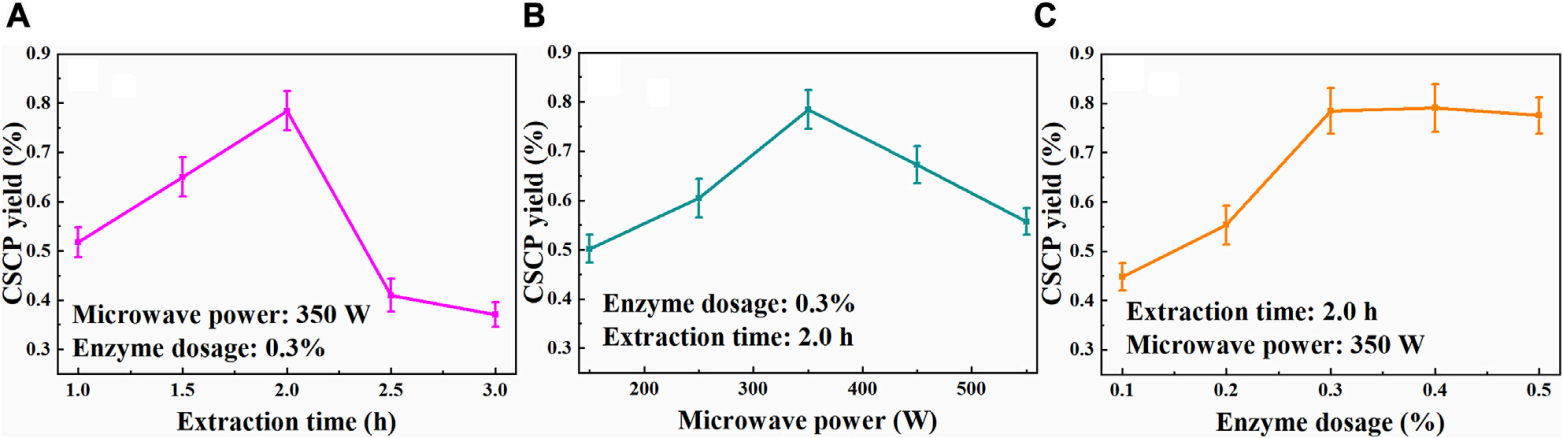
Effects of extraction time **(A)**, microwave power **(B)**, and enzyme dosage **(C)** on the CSCP yields.

Extraction time is a vital factor that could significantly affect the yield of CSCP. The effect of the extraction time on CSCP yield was evaluated and is shown in Figure 1A, while other extraction conditions were fixed at temperature 50°C, enzyme loading doses 0.3%, and microwave power 350 W. It was observed that the extraction yield of CSCP increased with the extending of the extraction time from 1.0 to 2.0 h, and then decreased when the extraction time further prolonging. A maximum CSCP yield of 0.78% was achieved at 2.0 h. This phenomenon maybe attributed to that the extracted polysaccharides could be hydrolyzed according to the destruction of spatial structure and the cleavage of chemical bonds with the time prolonging, leading to the decline in CSCP yields.

The effect of microwave power on the polysaccharides yield during the extraction process was also explored. The CSCP yields under different microwave power at 50°C for 2.0 h with 0.3% enzyme dosage are shown in Figure 1B. As expected, the CSCP extraction yield increased with the enhancement of the microwave power from 150 to 350 W, while it decreased as the microwave power continued to rise (350–550 W). It suggested that the higher microwave power could promote the mass transfer of *Chlorella* sp. cell, yielding the increase of the CSCP extraction yield. Microwave heating as a volumetrically distributed heat source was generated by ionic conduction of dissolved ions and dipole rotation of polar solvent, which leads to the effective cell rupture, releasing the polysaccharides into the solvent (Yuan et al., 2018). However, the degradation of the extracted CSCP occurred when the microwave power was further elevated from 350 to 550 W. Hence, microwave power of 350 W was selected for the further optimization.

Based on the above experimental results, the effect of enzyme dosage on CSCP yield was evaluated at 50°C for 2.0 h with microwave power of 350 W. As shown in Figure 1C, the dissolution of polysaccharides increased with the continuous addition of enzyme, and a maximum extraction yield of 0.78% was achieved when 0.3% of enzyme concentration was used. The CSCP yield remained stable when enzyme dosage further increased, and further increase of enzyme dosage just slightly increased the CSCP yield. The amount of substrate was too small compared to the enzyme dosage, and a large number of enzymes could not fully contact the substrate, so the polysaccharides extraction yield remained stable as the increase of enzyme loading. In the single-factor experiment, 0.3% of enzyme dosage was suitable for the polysaccharides extraction from *Chlorella* sp. biomass. e Silva et al. (2018) studied the polysaccharides extraction from microalgae *Arthrospira platensis* using water as a solvent, and 127 ± 5 mg of carbohydrate/g of biomass was obtained in a microwave power of 434 W for 1 min, biomass:solvent ratio 1:30 w·v^−1^. In addition, MAEE method had been proved to be efficient for extracting polysaccharides from *S. chinensis* baill, mountain *Zizania latifolia* swollen culm, *Sagittaria trifolia* tuber, et al. (Cheng et al., 2015; Zhang et al., 2022; Zhang et al., 2023).

#### 3.1.2 Model establishment and variance analysis

Based on the single-factor experiment, three experimental factors (enzyme dosage, microwave power and extraction time) were selected as the independent variables of BBD. The experimental design results are shown in Table 3. Design Expert 8.0 was used to perform quadratic multiple regression fitting analysis on the experimental design results and obtain the quadratic multiple regression equation:

$$Y=0.76+0.086A+0.015B+0.013C-0.025AB-0.033AC-0.124BC-0.09A^{2}-0.15B^{2}-0.07C^{2}$$

**TABLE 3:** Optimization of CSCP extraction conditions by Box-Behnken experimental design with the independent variables.

Run	A^a^	B	C	Yield of CSCP (%)
1	0	0	0	0.76
2	0	−1	−1	0.44
3	0	0	0	0.77
4	0	−1	1	0.66
5	0	0	0	0.78
6	−1	−1	0	0.38
7	1	0	−1	0.68
8	1	−1	0	0.61
9	−1	1	0	0.49
10	−1	0	−1	0.45
11	0	1	−1	0.69
12	−1	0	1	0.60
13	0	0	0	0.76
14	1	1	0	0.62
15	0	0	0	0.75
16	0	1	1	0.41
17	1	0	1	0.70

^a^ A: enzyme dosage; B: microwave power; C: extraction time.

The variance results of the experimental design analysis are shown in Table 4. It was found that the response surface regression model is extremely significant (*p*-value <0.0003). The lack of fit item (*p*-value >0.05) is not significant, suggesting that the nonexperimental factors have little effect on the results of the experimental data. A good relationship between regression model and actual extraction operation illustrates that the model is suitable for the prediction of polysaccharides extraction. The coefficient of determination (*R* ^2^) is 0.9650, which indicates that 96.50% of the variability in the response value can be explained by the model. The coefficient of determination after correction of the model is R^2^_Adj_, and the R^2^_Adj_ value of 0.9201 shows that the adjusted model has relatively high accuracy and versatility. Therefore, the equation obtained by the regression model can be applied to the analysis and prediction of the extraction process of CSCP. Based on the *p*-value, the linear variable A and the quadratic variables A^2^ and B^2^ had extremely significant effects on the extraction of CSCP (*p*-value <0.002). The two-variable interaction BC had significant effect (*p*-value = 0.0004) on CSCP yield. In addition, the influence of factors on the test results can be predicted according to the size of the *F* value. The influence of the test factors on the test results is as follows: enzyme dosage > microwave power > extraction time. Three experimental factors (enzyme dosage, microwave power and extraction time) all played key role in the extraction yield of CSCP.

**TABLE 4:** Analysis of variance for the extraction yield of CSCP.

Source	Sum of squares	Degree of freedom	Mean square	*F* Value	*p*-Value
Model	0.2893	9	0.0321	21.47	0.0003
A	0.0593	1	0.0593	39.57	0.0004
B	0.0018	1	0.0018	1.20	0.3088
C	0.0013	1	0.0013	0.8857	0.3780
AB	0.0024	1	0.0024	1.62	0.2433
AC	0.0044	1	0.0044	2.96	0.1291
BC	0.0615	1	0.0615	41.09	0.0004
A^2^	0.0348	1	0.0348	23.22	0.0019
B^2^	0.0913	1	0.0913	60.99	0.0001
C^2^	0.0180	1	0.0180	11.99	0.0105
Residual error	0.0105	7	0.0015		
Mismatch items	0.0099	3	0.0033	21.93	0.0060
Pure error	0.0006	4	0.0002		
Summation	0.2998	16			

Note: *p* < 0.01, difference was extremely significant; *p* < 0.05, significant difference; *R* ^2^ = 0.9650; R^2^_Adj_ = 0.9201.

#### 3.1.3 Interactive trial analysis

**FIGURE 2:**
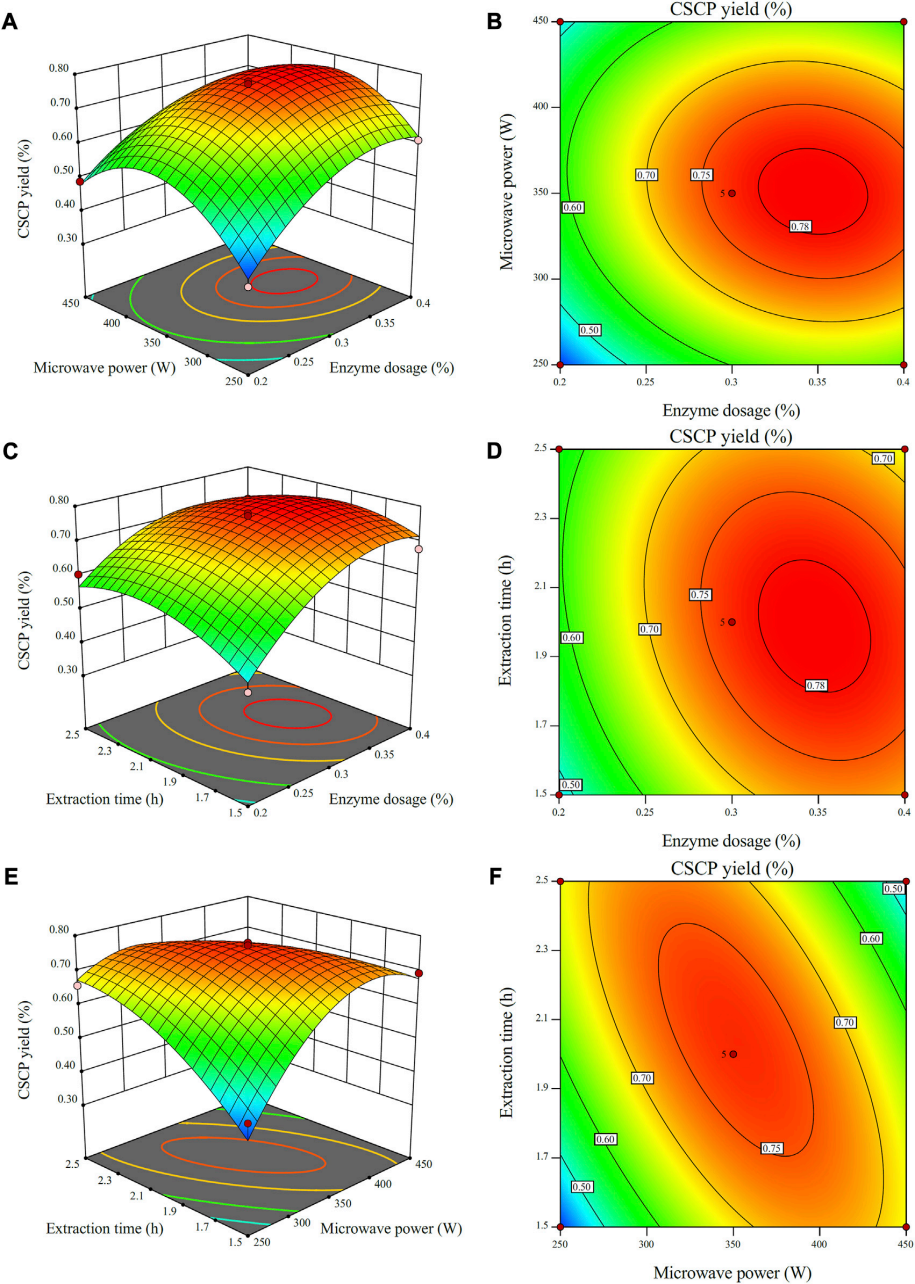
Response surface plots **(A,C,E)** and contour plots **(B,D,F)** for the effects of independent variables (extraction time, microwave power, and enzyme dosage) on the yields of CSCP.

To uncover the integrated effects of two operational parameters on the CSCP yield, a three-dimensional analysis of the regression model was operated by fixing one factor at the zero level. The interactive influence of enzyme dosage, microwave power and extraction time on CSCP yield is shown in Figures 2A–F. It was reported that the steeper the slope in response surface, the greater the relationship between independent variables and CSCP yield (Bankole et al., 2021; Ebrahimi et al., 2021). Conversely, the slope of the three-dimensional effect map in response surface is gentler, which demonstrates that the relationship between response value and variables is smaller, and the interactive influence of the two factors on CSCP yield is limited.

As shown in Figure 2A, the extraction yield of the CSCP increased with the enhance of enzyme dosage and microwave power when extraction time was artificially fixed at zero. The maximum value of CSCP extraction yield was also observed from the response surface plot. The interactive effect of enzyme dosage and microwave power on CSCP yield presented a significant interaction. In the contour line diagram of the model analysis result of the plane, the more elliptical the contour line effect is, the greater the relationship between independent variables and CSCP yield, and the more significant the interactive effect of the two factors. As shown in Figure 2D, the contour lines are roughly elliptical and densely distributed, indicating that the interactive effect of enzyme dosage and extraction time was also significant. As shown in Figure 2F, the contour lines are closer to elliptical as compared to that in Figures 2B, D, illustrating the strong interactive effect from microwave power and extraction time.

### 3.2 Single-factor experiments for enzyme-modified polysaccharides

**FIGURE 3:**
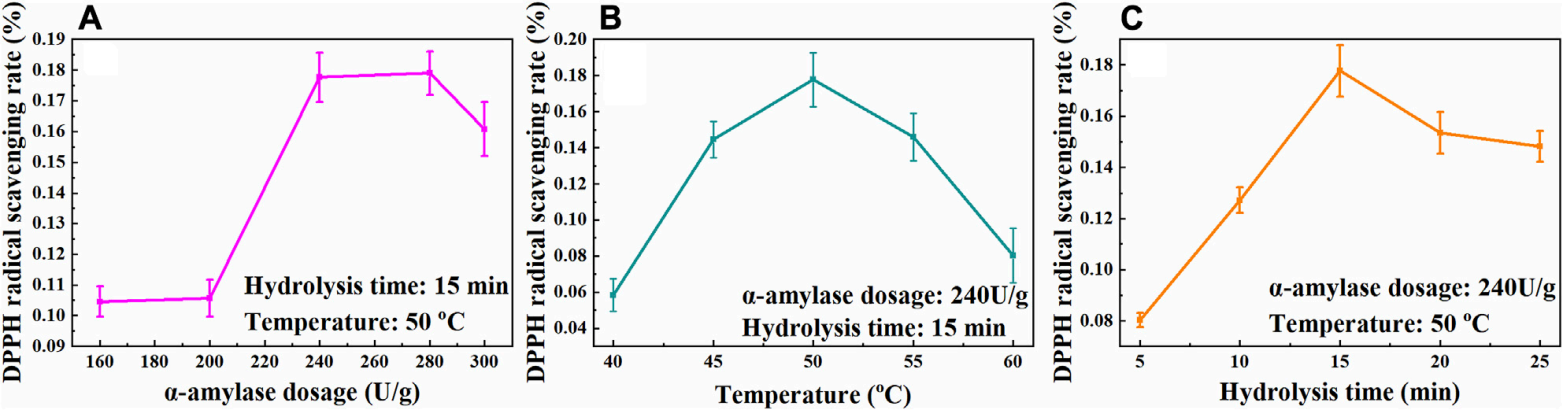
Effects of α-amylase dosage **(A)**, temperature **(B)**, and hydrolysis time **(C)** on the scavenging rate of DPPH free radicals.

Polysaccharides are biological macromolecules with many functional groups encapsulated within the structure. Spatial structural modification of the polysaccharides could expose these groups and improve their biological activity (Huang et al., 2020; Ke and Li, 2022). In biological methods, polysaccharides are usually treated by enzymes, which are divided into hydrolase and oxidase. Hydrolase can hydrolyze polysaccharides, reduce their molecular weight and interfacial tension. In the process of enzymatic hydrolysis, enzymes could break the bonds between polysaccharides, exposing more functional groups of polysaccharides, thus promoting their biological activity and achieving the purpose of enzyme modification (Tang and Huang, 2022). The effects of enzyme-modified temperature, α-amylase dosage and hydrolysis time on the bioactivity of the CSCP were studied. The optimal enzyme-modified conditions were evaluated via antioxidant activity of the modified CSCP by determining the 1,1-diphenyl-2-picrylhydrazyl (DPPH) radical scavenging rate. As is shown in Figure 3, it was observed that the DPPH radical scavenging rate of the modified CSCP reached a maximum of 17.77% when α-amylase dosage was 240 U/g at 50°C for 15 min.

#### 3.2.1 Model establishment and variance analysis

The experimental design results are shown in Table 5. Design Expert 8.0 was used to perform quadratic multiple regression fitting analysis on the experimental design results and obtain the quadratic multiple regression equation:

$$Y=17.72+2.53A+1.29B+0.2113C-1.96AB-1.04AC-1.11BC-2.94A^{2}-2.57B^{2}-2.30C^{2}$$

**TABLE 5:** Box-Behnken experimental design with the independent variables for optimizing the conditions of enzymatic modification of CSCP.

Run	A^a^	B	C	Scavenging rate of DPPH radical (%)
1	0	1	1	13.45
2	0	0	0	17.77
3	−1	−1	0	5.61
4	−1	0	−1	9.73
5	0	0	0	17.81
6	0	0	0	17.65
7	1	0	−1	15.64
8	0	−1	−1	10.04
9	1	−1	0	15.82
10	0	1	−1	14.41
11	0	0	0	17.68
12	0	0	0	17.71
13	−1	1	0	12.51
14	−1	0	1	11.40
15	1	1	0	14.90
16	0	−1	1	13.51
17	1	0	1	13.15

^a^ A: α-amylase dosage; B: hydrolysis time; C: temperature.

The variance results of the experimental design results analysis are shown in Table 6. Among them, the response surface regression model is extremely significant (*p*-value <0.0001). The lack of fit item (*p*-value >0.05) is not significant, illustrating that the nonexperimental factors have little effect on the results of the experimental data, and the regression model has a good relationship with the actual enzyme-modified conditions. The model determination coefficient (*R* ^2^ = 0.9748) indicates that 97.48% of the change in the response value can be explained by the model. Therefore, the equation obtained by the regression model is within the experimental range and can be fully applied to the analysis and prediction of the modification process of CSCP. From the data in Table 6, it can be concluded that the influence of the test factors on the test results follows the order: enzyme dosage > hydrolysis time > enzyme-modified temperature.

**TABLE 6:** Analysis of variance for the optimization of enzyme modified polysaccharide conditions.

Source of variance	Quadratic sum	Degree of freedom	Mean square	F Ratio	P Ratio
Model	186.05	9	20.67	30.13	<0.0001
A	51.31	1	51.31	74.79	<0.0001
B	13.24	1	13.24	19.29	0.0032
C	0.3570	1	0.3570	0.5204	0.4941
AB	15.29	1	15.29	22.28	0.0022
AC	4.33	1	4.33	6.31	0.0403
BC	4.91	1	4.91	7.15	0.0318
A^2^	36.47	1	36.47	53.17	0.0002
B^2^	27.83	1	27.83	40.56	0.0004
C^2^	22.29	1	22.29	32.49	0.0007
Residual error	4.80	7	0.6860		
Mismatch items	4.79	3	1.60	372.68	<0.0001
Pure error	0.0171	4	0.0043		
Summation	190.85	16			

Note: *p* < 0.01, difference was extremely significant; *p* < 0.05, significant difference; *R* ^2^ = 0.9748; R^2^_Adj_ = 0.9425.

#### 3.2.2 Interactive trial analysis

**FIGURE 4:**
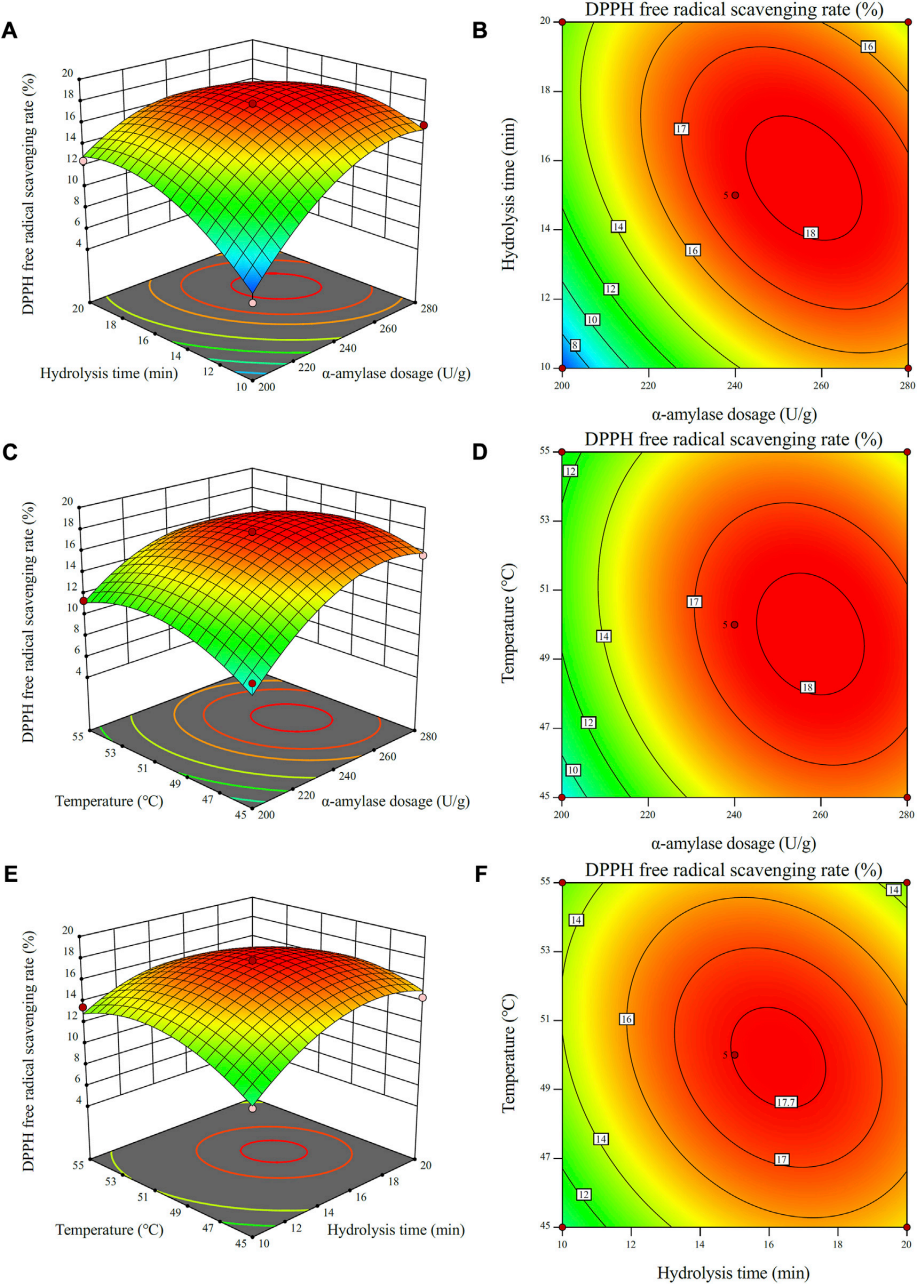
Response surface plots **(A,C,E)** and contour plots **(B,D,F)** for the effects of independent variables (α-amylase dosage, temperature, and hydrolysis time) on the scavenging rate of DPPH free radicals.

The interactive influence of enzyme dosage, enzyme-modified temperature and hydrolysis time on antioxidant activity of the CSCP was evaluated by a three-dimensional analysis of the regression model (Figures 4A–F). As shown in Figure 4A, the DPPH radical scavenging rate of the CSCP increased with the increase of the α-amylase dosage and hydrolysis time when enzyme-modified temperature was fixed at zero. A maximum value of the DPPH radical scavenging rate was achieved and a high point was presented on the surface. Clearly, the interaction between the enzyme dosage and the hydrolysis time was extremely significant. As shown in Figures 4D, F, the contour lines are relatively close to circular, indicating the weak influence from enzyme-modified temperature with time and enzyme dosage, which is also consistent with the analysis in Table 6.

### 3.3 Validation of predicted models

Based on the response surface analysis, the optimal condition for the extraction of CSCP was at 50°C for 2.31 h with 379.24 W of microwave power and 0.31% of enzyme dosage. Under the experimental condition, the predicted extraction yield of CSCP was 0.72%. To validate the accuracy of the model equation, confirmatory experiment was conducted at 50°C for 2.3 h with 380 W of microwave power and 0.31% of enzyme dosage. The extraction yield of CSCP (0.72% ± 0.02%) was close to the predicted value, which indicated that the regression model was suitable for the prediction of CSCP extraction yield.

The regression model of the enzyme modification experiment was predicted by the response surface design, and the optimal modification condition was obtained as follows: enzyme dosage 270.99 U/g, hydrolysis time 14.02 min, and enzyme-modified temperature 51.33°C. Under this condition, the scavenging rate of DPPH free radicals reached 17.60%. Considering the actual test condition, the adjusted optimal condition was as follows: an enzyme dosage of 271 U/g, a modification time of 14 min, and a modification temperature of 51°C. Under the condition, the modification effect of CSCP was verified by multiple tests, and the scavenging rate of DPPH free radicals was 17.58% ± 0.1%, which was close to the above predicted value, indicating that the regression model could be used to optimize the α-amylase modification of CSCP.

### 3.4 Characterization of CSCP

**FIGURE 5:**
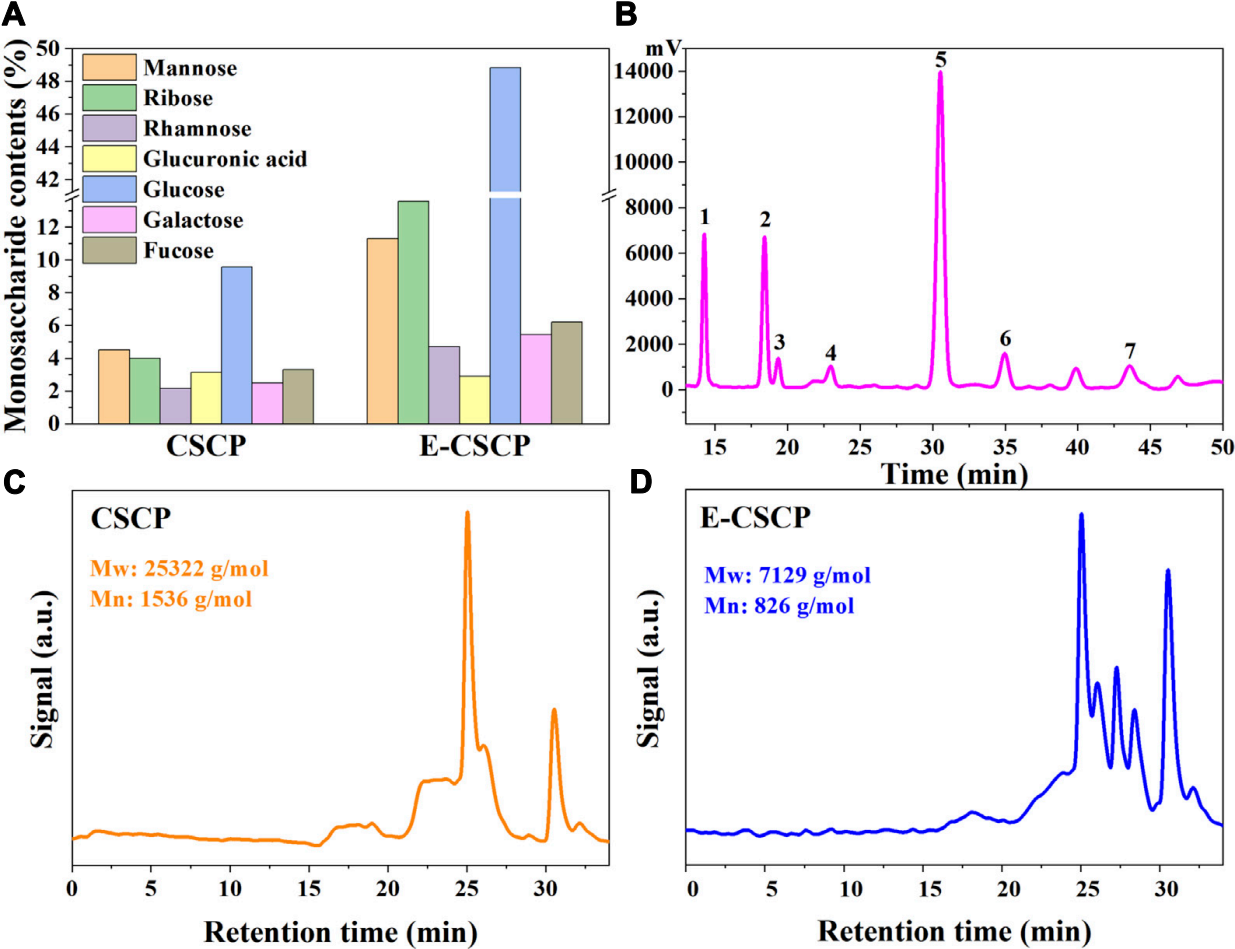
**(A)** The monosaccharide contents of CSCP and E-CSCP, **(B)** The monosaccharide compositions of E-CSCP, Peaks 1. Mannose, 2. Ribose, 3. Rhamnose, 4. Glucuronic acid, 5. Glucose, 6. Galactose, and 7. Fucose, Molecular weight distribution of samples **(C)** CSCP and **(D)** E-CSCP.

The monosaccharide compositions of crude CSCP and enzyme-modified CSCP (E-CSCP) were evaluated and illustrated in Figures 5A, B. It was observed that the total monosaccharides content in crude CSCP was only 29.15%, which may be ascribed to that the sugars in *Chlorella* sp. were mostly separated in the form of polysaccharides under the optimal extraction condition. After enzyme modification, the monosaccharides content in E-CSCP increased dramatically from 29.15% to 93.51%, indicating that the enzyme modification process promoted the disconnection of the bonds between polysaccharides, thus enhancing the biological activity of E-CSCP. The composition analysis of E-CSCP presented that glucose (48.84%), ribose (13.57%) and mannose (11.30%) were the major compositional monosaccharides, while a small quantity of fucose (6.21%), galactose (5.44%), rhamnose (4.71%) and glucuronic acid (2.89%) were also detected. Additionally, the protein and pigment in the extracted CSCP were removed, and the removal rates for protein and pigment were 15.18% and 77.15%, respectively.

As shown in Figures 5C, D the weight-average (M_w_) and number-average (M_n_) molecular weights of crude CSCP and E-CSCP were analyzed by gel permeation chromatography (GPC). The M_w_ and M_n_ for E-CSCP were 7,129 g/mol and 826 g/mol, respectively, which exhibited a significant decrease as compared to that of the crude CSCP (M_w_, 25,322 g/mol, M_n_, 1,536 g/mol). It can be inferred that enzyme modification of the CSCP promoted the cleavage of bonds, reducing the molecular weight (Peng et al., 2022).

**FIGURE 6:**
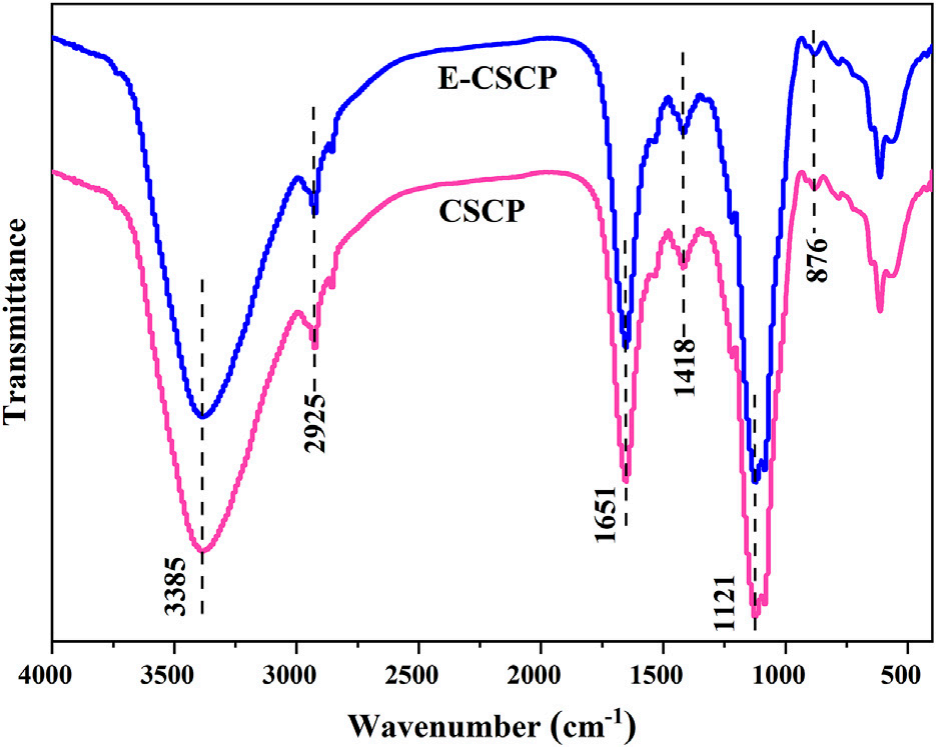
FT-IR spectra of CSCP and E-CSCP.

To further identify the functional groups in the structure of CSCP and E-CSCP, FT-IR analysis was also operated and is shown in Figure 6. The peak at 3,385 cm^-1^ is ascribed to the signal from the -OH stretching. The absorption band centered at 2,925 cm^-1^ is the C-H stretching vibration (Zhuang et al., 2019). The peaks at 1,651–1,418 cm^-1^ are assigned to the signals of ester carbonyl groups (C=O) and carboxylic groups (COO-), illustrating the presence of uronic acids in CSCP. The band observed at 1,121 cm^-1^ is assigned to the signal from pyranose ring. The peak at 876 cm^-1^ is related to the glycosidic linkage vibrations (Vázquez-Vuelvas et al., 2020). Therefore, the FT-IR spectra from sample CSCP and E-CSCP suggested that the extracted saccharides from *Chlorella* sp. presented typical absorption peaks of polysaccharides.

## 4 Conclusion

The extraction conditions and α-amylase modification conditions of CSCP were optimized by BBD and RSM. The CSCP yield of 0.72% was obtained under the follow conditions: microwave power 380 W, enzyme dosage 0.31%, pretreatment time 2.3 h. The optimal conditions for enzymatic modification of CSCP were also optimized, in which the DPPH radical scavenging rate was used as the response value. The scavenging rate of DPPH free radicals was 17.58% when enzyme dosage was 271 U/g at 51°C for 14 min. The scavenging rate of DPPH free radicals reached 17.58%, suggesting that the modification method and conditions could effectively improve the biological activity of CSCP. In addition, the enzyme-modified CSCP presented a typical heteropolysaccharide mainly including glucose, ribose and mannose, which has good potential as a natural antioxidant used in the food or medicine industry.

## Data Availability

The original contributions presented in the study are included in the article/Supplementary Material, further inquiries can be directed to the corresponding authors.
